# Supplementary material for: Towards a More Efficient Breast Cancer Therapy Using Active Human Cell Membrane-Coated Metal–Organic Frameworks
Source: Nanomaterials (Basel). 2024 Apr 30;14(9):784. doi: 10.3390/nano14090784 (PMC11085653; doi:10.3390/nano14090784)
Supplement: Supplementary file 1 [file nanomaterials-14-00784-s001.zip › nanomaterials-2981197-supplementary.pdf]

Supporting information

Towards a More Efficient Breast Cancer Therapy Using Active Human Cell Membrane-Coated Metal–Organic Frameworks

Pablo Graván [1,2,3,4,5,6], Sara Rojas [7], Darina Francesca Picchi [8,9], Francisco Galisteo-González [1], Patricia Horcajada [8,*] and Juan Antonio Marchal [2,3,4,5,6,*]

[1] Department of Applied Physics, Faculty of Science, University of Granada, 18071 Granada, Spain; gravan@ugr.es (P.G.); galisteo@ugr.es (F.G.-G.)
[2] Department of Human Anatomy and Embryology, Faculty of Medicine, University of Granada, 18016 Granada, Spain
[3] Instituto de Investigación Biosanitaria de Granada (ibs. GRANADA), 18012 Granada, Spain
[4] Biopathology and Regenerative Medicine Institute (IBIMER), Centre for Biomedical Research (CIBM), University of Granada, 18016 Granada, Spain
[5] Excellence Research Unit Modelling Nature (MNat), University of Granada, 18016 Granada, Spain
[6] BioFab i3D—Biofabrication and 3D (Bio)Printing Laboratory, University of Granada, 18100 Granada, Spain
[7] Department of Inorganic Chemistry, Faculty of Science, University of Granada, Av. Fuentenueva s/n, 18071 Granada, Spain; srojas@ugr.es
[8] Advanced Porous Materials Unit, IMDEA Energy Institute, Av. Ramón de la Sagra 3, 28935 Móstoles, Spain; darina.picchi@imdea.org
[9] Escuela Internacional de Doctorado, Universidad Rey Juan Carlos, c/Tulipan, s/n, Móstoles, 28933 Madrid, Spain
* Correspondence: patricia.horcajada@imdea.org (P.H.); jmarchal@ugr.es (J.A.M.)

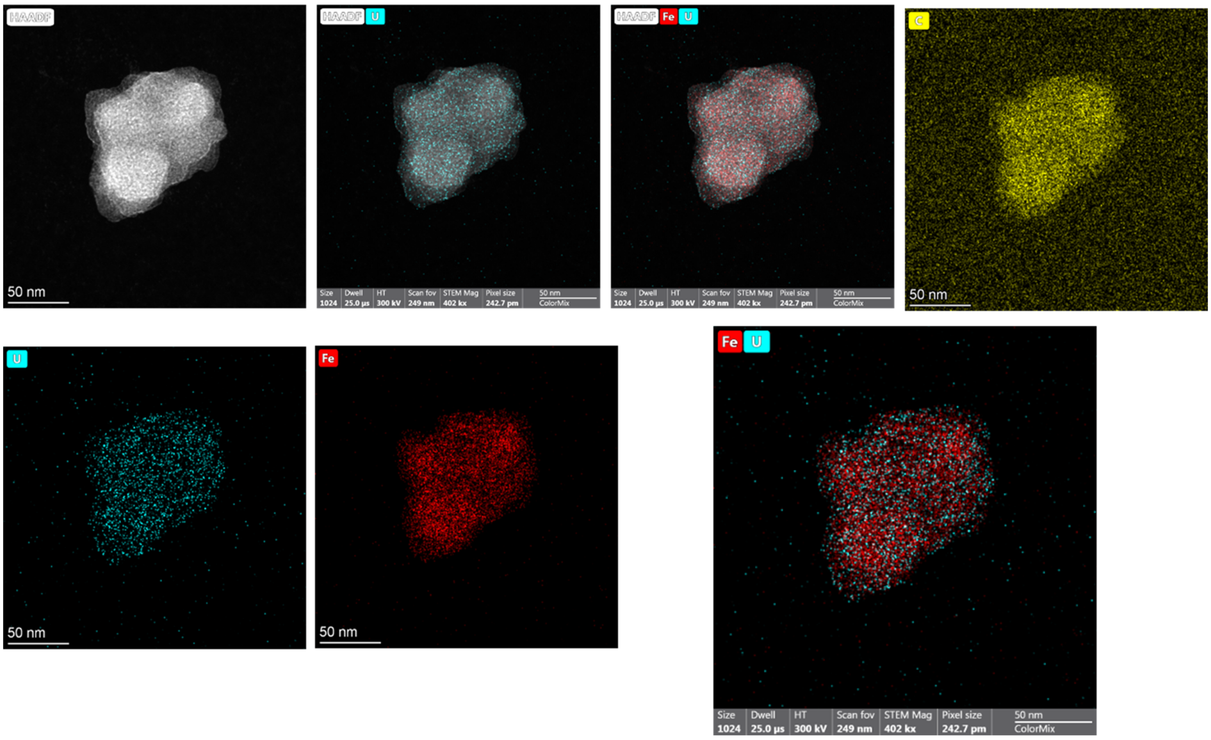

Figure S1. HR-TEM images and EDX-elemental mapping confirming the successful cell membrane coating of MIL-100(Fe) NPs (Fe: red, U: blue, C: yellow).

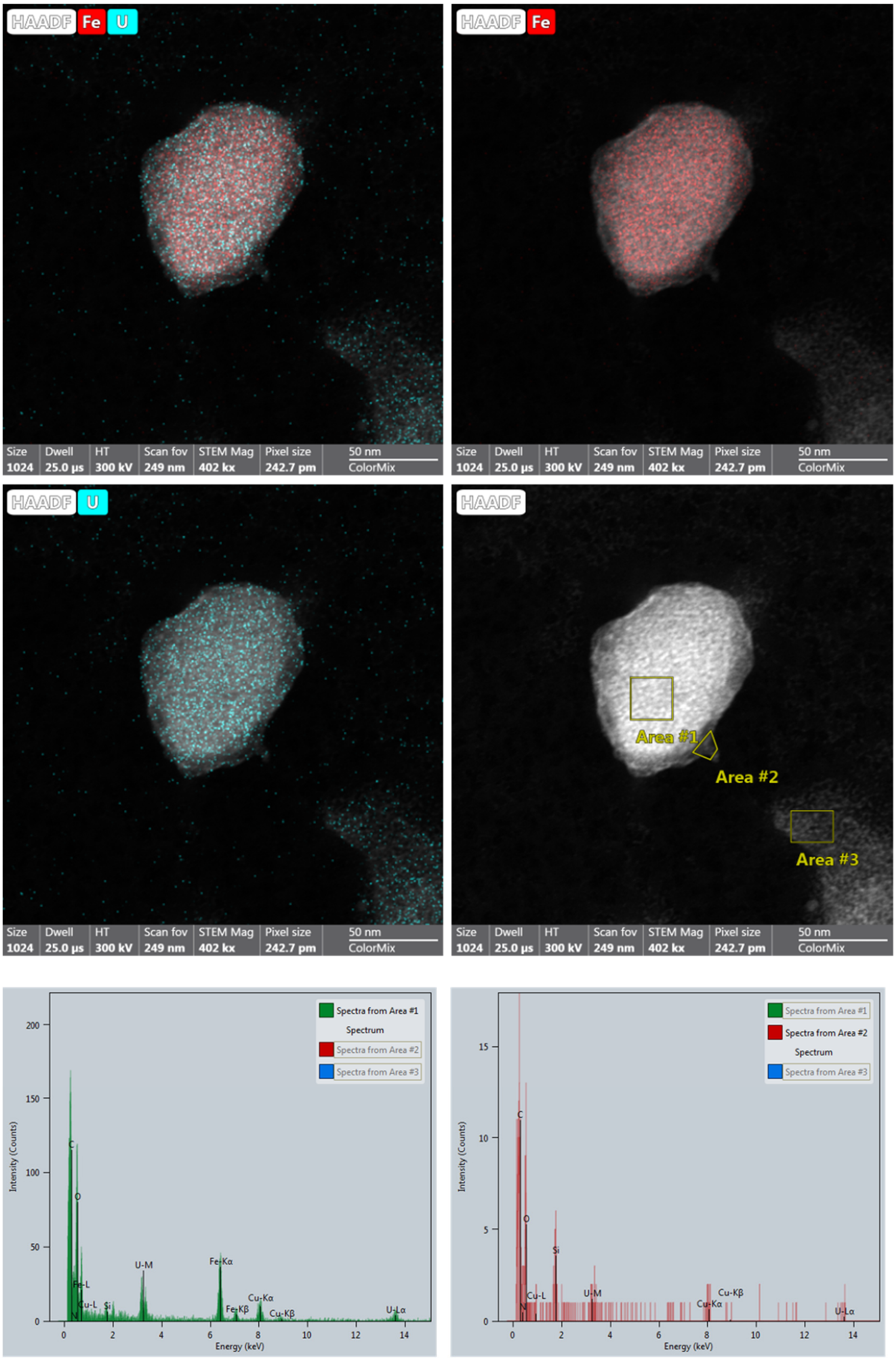

Figure S2. HR-TEM images and EDX-elemental mapping/counts of internal and external area of CS_MIL-100(Fe) NPs confirming differences in the Fe:U ratio (Fe: red, U: blue, C: yellow).

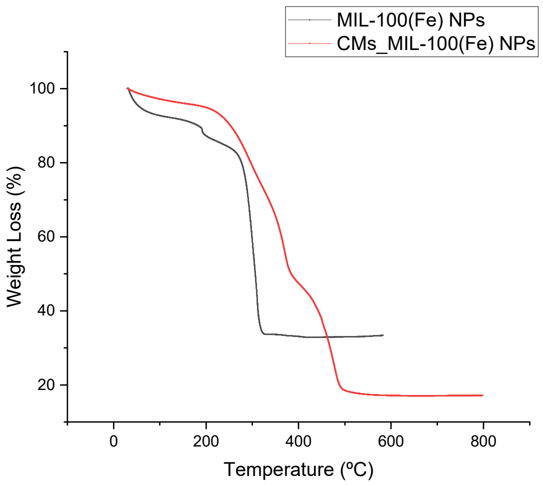

Figure S3. TGA trace for MIL-100(Fe) (black) and CMs_MIL-100(Fe) NPs (red).
